# Fitness Trade-offs Restrict the Evolution of Resistance to Amphotericin B

**DOI:** 10.1371/journal.pbio.1001692

**Published:** 2013-10-29

**Authors:** Benjamin Matteson Vincent, Alex Kelvin Lancaster, Ruth Scherz-Shouval, Luke Whitesell, Susan Lindquist

**Affiliations:** 1 Microbiology Graduate Program, Massachusetts Institute of Technology, Cambridge, Massachusetts, United States of America; 2 Whitehead Institute for Biomedical Research, Cambridge, Massachusetts, United States of America; 3 Howard Hughes Medical Institute, Department of Biology, Massachusetts Institute of Technology, Cambridge, Massachusetts, United States of America; Carnegie Mellon University, United States of America

## Abstract

The evolution of drug resistance in microbial pathogens provides a paradigm for investigating evolutionary dynamics with important consequences for human health. *Candida albicans*, the leading fungal pathogen of humans, rapidly evolves resistance to two major antifungal classes, the triazoles and echinocandins. In contrast, resistance to the third major antifungal used in the clinic, amphotericin B (AmB), remains extremely rare despite 50 years of use as monotherapy. We sought to understand this long-standing evolutionary puzzle. We used whole genome sequencing of rare AmB-resistant clinical isolates as well as laboratory-evolved strains to identify and investigate mutations that confer AmB resistance *in vitro*. Resistance to AmB came at a great cost. Mutations that conferred resistance simultaneously created diverse stresses that required high levels of the molecular chaperone Hsp90 for survival, even in the absence of AmB. This requirement stemmed from severe internal stresses caused by the mutations, which drastically diminished tolerance to external stresses from the host. AmB-resistant mutants were hypersensitive to oxidative stress, febrile temperatures, and killing by neutrophils and also had defects in filamentation and tissue invasion. These strains were avirulent in a mouse infection model. Thus, the costs of evolving resistance to AmB limit the emergence of this phenotype in the clinic. Our work provides a vivid example of the ways in which conflicting selective pressures shape evolutionary trajectories and illustrates another mechanism by which the Hsp90 buffer potentiates the emergence of new phenotypes. Developing antibiotics that deliberately create such evolutionary constraints might offer a strategy for limiting the rapid emergence of drug resistance.

## Abstract 2

The rarity of clinical drug resistance to the antifungal amphotericin B is explained by the extreme costs that resistance mutations impose upon stress responses and virulence factors.

## Author Summary

The evolution of drug resistance in human pathogens is considered an inevitable consequence of the selective pressures imposed by antimicrobial drugs. Yet resistance to one antifungal drug, amphotericin B (AmB), remains extremely rare despite decades of widespread use. Here we explore the biological mechanisms underlying this conundrum. By examining natural and experimental populations of *Candida albicans*, we identify multiple mutations that confer resistance to AmB *in vitro*. As with the evolution of resistance to other antifungals, we find that the chaperone protein Hsp90 is involved in enabling the evolution of resistance to AmB. We also discover, however, that mutations that confer AmB resistance impose massive costs on other aspects of fungal pathogenicity; strains that are resistant to AmB are hypersensitive to attack by the host immune system and are unable to invade and damage host tissue. Thus, the evolution of resistance to AmB is restricted by a tradeoff between tolerance of the drug and the ability to cause disease. We propose that developing new antibiotics for which resistance presents such dire tradeoffs may be a promising strategy to prevent the evolution of resistance.

## Introduction

Understanding how organisms rapidly evolve novel traits is a central problem in both evolutionary biology and the treatment of infectious diseases. The emergence of drug-resistant pathogens not only provides a model for studying the evolution of new phenotypes but also poses a grave challenge to human health. Antibiotic treatment selects for rare mutations that alter cellular processes and, thereby, either mitigate the toxicity of the drug or bypass it altogether. Sometimes resistance mechanisms are completely orthogonal to the normal biology of the cell, as is the case for the amplification of efflux pumps or the horizontal acquisition of drug-detoxifying enzymes in bacteria. But often the mutations that confer resistance alter basic cellular processes in such a way as to create a variety of new stresses. The latter is especially relevant in eukaryotic pathogens, where the rarity of genetic exchange within and between populations necessitates the *de novo* evolution of resistance [1],[2].

*Candida albicans* is the leading fungal pathogen of humans and the fourth most common cause of all hospital-acquired infections [3],[4]. Normally a harmless commensal, changes in host immune status allow *C. albicans* to become pathogenic. Infections range from superficial thrush to life-threatening systemic disease. Mild to moderate infections are currently treated with triazoles, which inhibit Erg11 (lanosterol 14α-demethylase), preventing ergosterol biosynthesis [5]. Life-threatening systemic infections require treatment with echinocandin or polyene agents. Echinocandins inhibit the synthesis of the cell wall polymer (1,3)-β-D-glucan, resulting in loss of cell integrity [5].

The third most commonly employed antifungal is the polyene drug amphotericin B (AmB), which was the standard of care for ∼40 years [6]. Its potent fungicidal activity derives from its ability to selectively bind the major sterol of fungal membranes, ergosterol [7],[8]. Among other effects, this binding induces pore formation in the plasma membrane and results in rapid cell death. While AmB is extremely effective at killing fungi, its clinical utility is impaired by several liabilities. First, pharmacokinetics and distribution are poor, allowing some fungi to hide in niches where drug exposure is limited [9]. Second, AmB induces idiosyncratic systemic reactions involving fever and tremors. Third, and still more problematic, AmB's cumulative, dose-dependent renal toxicity limits use in many patients.

Despite these limitations, a remarkable advantage of AmB is that it has been exceptionally refractory to the evolution of resistance. After 50 years of use as monotherapy, the acquisition of AmB resistance in *C. albicans* remains extremely rare. For comparison, the antifungal drug 5-flucytosine was introduced several years later than AmB, but resistance rendered this drug obsolete against *Candida* in less than 20 years [10]. In a recent study of 9,252 clinical isolates of *C. albicans*, 99.8% remained AmB-sensitive [11]. Although the less toxic triazoles and echinocandins have recently replaced AmB as the frontline therapy, AmB still retains frequent use in many settings, particularly when an infection resists treatment with other drugs [12]. Indeed, resistance to triazoles emerges frequently and, although echinocandins are relatively new to the clinic, resistance to echinocandins is also already arising [13]–[18].

The best-validated mechanism of resistance to AmB observed in clinical isolates of *C. albicans* to date involves a double loss of function in both *ERG3* and *ERG11* (C-5 sterol desaturase and lanosterol 14α-demethylase, respectively), identified by biochemical analysis of membrane sterol composition [19]–[21]. In other fungal pathogens, sterol analysis of rare AmB-resistant isolates has identified resistant strains lacking *ERG2*, encoding C-8 sterol isomerase, and *ERG6*, encoding C-24 sterol methyltransferase [22]–[24]. However, there has been no systematic analysis of AmB resistance mutations in *Candida* using matched isogenic strains. More importantly, the consequences of these mutations upon the biology and pathogenicity of *Candida* remain largely unexplored.

Here we thoroughly explore mutations that can confer AmB resistance in *C. albicans* with the goal of understanding why resistance emerges so rarely in the clinic. Our results establish that the evolutionary constraints imposed by AmB are distinct from those of other antifungals. They provide insights into the mechanisms by which external and internal biological stresses restrict evolutionary trajectories.

In addition, our work broadens the role of protein homeostasis regulators as potentiators for the emergence of new traits. Finally, our findings suggest a general strategy for antimicrobial drug development that might be broadly useful in limiting the emergence of resistance.

## Results

### Whole Genome Sequencing of AmB-Resistant Clinical Isolates implicates *ERG2* and *ERG3/11*

As a first step towards understanding the evolution of resistance to AmB in *Candida*, we sought to broaden and validate the list of mutations that allow the fungus to tolerate this drug. As AmB-sensitive parental strains from which rare AmB-resistant isolates evolved are not available, the identification of mutations conferring resistance has proven challenging. Nevertheless, we sequenced the entire genome of two independent clinical isolates that had evolved resistance to AmB, one from *C. albicans* and one from the closely related species *C. tropicalis*. For comparison, we also resequenced the AmB-sensitive *C. albicans* reference strain SC5314.

Using paired-end reads, we achieved over 50-fold coverage of these genomes, which allowed us to detect simple polymorphisms as well as complex genome rearrangements. As expected, the strains differed from each other and from the reference strain at more than 20,000 sites. To identify the variants responsible for resistance, we took advantage of previous work and inspected candidate genes acting in the ergosterol biosynthesis pathway.

**Figure 1:**
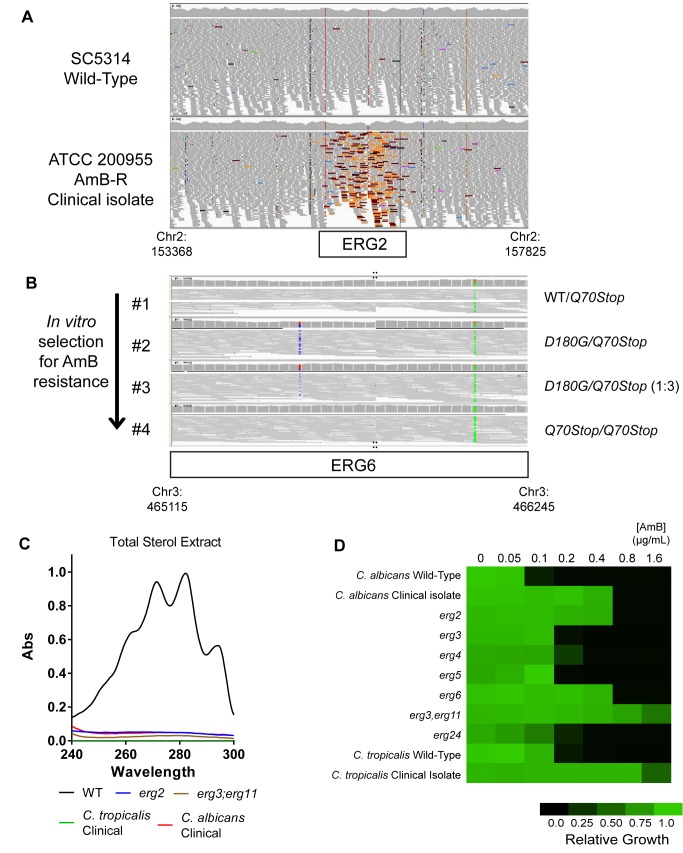
Mechanisms of AmB resistance in *Candida*. (A) Alignment of reads from whole-genome sequencing of *C. albicans* wild-type strain SC5314 and AmB-resistant clinical isolate ATCC 200955 demonstrates transposon insertion in *ERG2* in the clinical isolate. The *ERG2* locus is shown. Colored reads are indicative of mate-pairs that do not both map to the same chromosome, but instead one end to ERG2 and the other end to the TCA2 locus (elaborated in Figure S1A). Reads were visualized with the integrative genomics viewer (IGV) [53]. (B) Alignment of selected strains from whole-genome sequencing of *in vitro*–evolved AmB-resistant series identifies causal mutations. Mutations in *ERG6* ORF are highlighted, and the corresponding amino acid changes are indicated. Strain #1, 0 generations (founder); Strain #2, 60 generations; Strain #3, 120 generations; Strain #4, 240 generations. Two segments of IGV visualization for ERG6 were joined to allow visualization of both mutations in one image; point of joining indicated by “::”. (C) Spectrophotometric analysis of sterols reveals lack of C5–C6∶C7–C8 conjugation in AmB-resistant clinical isolates as well as laboratory-generated *erg2* and *erg3 erg11* mutants. Sterols were isolated by saponification and heptane extraction and analyzed spectrophotometrically between 240 and 300 nm, following established methods [55]. (D) AmB susceptibility of clinical isolates and laboratory-generated mutants in every nonessential gene in the latter half of the ergosterol biosynthesis pathway (after cyclization of squalene to lanosterol). Mutants were generated in SN152 strain background using HIS1, LEU2, and ARG4 markers [56]. AmB susceptibility was determined by microplate dilution in RPMI at 37°C for 24 h, repeated in duplicate; growth was normalized to wild-type in the absence of AmB.

In the *C. albicans* AmB-resistant isolate, we detected a high density of mispaired reads at the *ERG2* (*ORF19.6026*) locus (Figure 1A). Further analysis revealed that both copies of the *ERG2* gene in this strain carried an insertion of the TCA2 retrotransposon (Figure S1A). Whole-genome analysis of polymorphisms indicated that the strain carried a high level of heterozygosity across its entire genome, with only two small regions of homozygosity. Strikingly, one of these included the transposon insertion in *ERG2* (Figure S1B).

In the *C. tropicalis* isolate, the sequence of *ERG2* was identical to that of the AmB-sensitive reference strain, MYA-3404. However, a mutation was observed in *ERG3* (*CTRG_04480*), another enzyme involved in sterol synthesis (Figure S2A). Specifically, phenylalanine replaced serine 258, a residue that is absolutely conserved in this protein from fungi to mammals (Figure S2B). In addition, the *ERG11* (*CTRG_05283*) ORF of this isolate harbored a deletion of 170 nucleotides (Figure S2C). Again, despite generally high levels of heterozygosity in other regions of the genome, the regions surrounding both *ERG3* and *ERG11* had become homozygous (Figure S2D). These results suggest that selective sweeps had operated to fix new mutations to a homozygous state in both clinical isolates. But, of course, the sequencing of many more AmB-sensitive and resistant isolates would be necessary to establish this conclusion.

### Validation of *erg2* and *erg3/11* in Laboratory Strains

To validate that either loss of *ERG2* or the combined loss of *ERG3* and *ERG11* function is sufficient to confer resistance to AmB, we created them anew in a wild-type background. Because *C. albicans* is an obligate diploid, we used auxotrophic markers to sequentially delete the loci, creating homozygous mutations in *ERG2* and double homozygous mutations in *ERG3 and ERG11*.

To confirm the inactivation of these genes in both lab strains and clinical isolates, we exploited the unique spectral characteristics of ergosterol. These result from conjugation of the double bonds C5–C6 and C7–C8, formed and isomerized by Erg3 and Erg2, respectively. We prepared sterol extracts from both of the clinical isolates and both of the laboratory mutants along with the isogenic wild-type control. Extracts from the wild-type laboratory strain exhibited the double-peaked spectrum between 240 and 300 nm characteristic of the conjugated bonds (Figure 1C). Extracts from both the laboratory mutants and the clinical isolates did not.

Next, we determined the minimal inhibitory concentration (MIC) of AmB in the knockout strains and compared it to the wild-type laboratory strain and the clinical isolates. AmB resistance levels in the two newly created laboratory strains matched those of the corresponding clinical isolates. For the *erg2* mutants this was a 10-fold increase in MIC (Figure 1D), and for the *erg3 erg11* mutants it was a 20-fold increase. We were unable to obtain further transformants in these mutants for technical reasons, thus precluding any attempt at complementation of the phenotype. However, we verified our results with an additional independent mutant in each background (Figure S5). Thus, laboratory-generated mutants successfully reproduce the resistant phenotypes observed in clinical isolates.

### Laboratory Evolution of AmB Resistance Implicates *ERG6* Mutations

To discover other mutations that could confer AmB resistance in *Candida*, we next employed *in vitro* evolution. The drug-sensitive reference strain SC5314 was not suited to this analysis because we repeatedly found that the resistance that emerged in selections with this strain was highly unstable. Another strain, *C. albicans* ATCC-10231, proved to be less susceptible to this problem. To isolate resistant variants, this strain was inoculated in liquid media containing a low concentration of AmB and serially passaged seven times into media with a 2-fold higher concentration of AmB at each step. Surviving cells from each passage were isolated and saved. We then used whole genome sequencing and alignment of the parental strain with strains from the third, fifth, and seventh passages to identify the mutations emerging as strains developed resistance.

Alignment of genome sequences and algorithmic detection of novel polymorphisms emerging within the series revealed a trajectory of mutations in the *ERG6* (*ORF19.1631*) gene, encoding Δ(24)-sterol C-methyltransferase (Figure 1B). Notably, the parental strain had been heterozygous for a premature stop codon that replaced the codon for *Glu70*. A mutation in the other allele of *ERG6*, *Asp180Gly*, appeared at the second step in the series. In the third step, the copy number of the *Q70Stop* allele increased to a 3∶1 ratio relative to the *Asp180Gly* allele. Finally, a loss of heterozygosity event, unique to the left arm chromosome 3 where *ERG6* resides, resulted in homozygosity of the nonsense allele (Figure S3A–B).

Several independent selections with this strain—with either a similar gradual selection process or with selection regimes employing immediate shifts to high drug concentrations—all involved additional mutations in *ERG6* (unpublished data). Finally, we validated the capacity of *ERG6* mutations to create AmB resistance by using auxotrophic markers to delete the gene in the SC5314 reference strain background. This resulted in a 12-fold increase in the AmB MIC (Figure 1D).

### Systematic Analysis of AmB Resistance in Late-Stage Ergosterol Biosynthesis Mutants

To systematically define genes whose inactivation might confer AmB resistance *in vitro*, we used homologous site-directed recombination to generate isogenic diploid deletion mutants for all seven nonessential genes acting in the latter half of the ergosterol biosynthesis pathway (the steps after cyclization of squalene to lanosterol). Only the deletion of *ERG2*, *ERG6*, or of *ERG3* and *ERG11* together conferred more than a 3-fold increase in the AmB MIC (Figure 1D). While other mutations conferring resistance to AmB may exist, these three are the most critical, as they have all been detected in the clinic and validated in the laboratory. Thus, we focused our efforts to explain the exceptional rarity of clinical AmB resistance on understanding the broader biological consequences of mutations in *ERG2*, *ERG6*, or *ERG3* and *ERG11*.

### AmB-Resistant Mutants Have an Unusual and Extreme Dependence on Hsp90

We previously reported that the emergence and maintenance of resistance to triazole and echinocandin antifungals critically depends on the molecular chaperone Hsp90 (ORF19.6515) [25]. Hsp90 is one of the most abundant proteins in eukaryotic cells, constituting ∼1% of total cellular protein, and acts as a protein homeostasis buffer. Although Hsp90 is an essential protein, its activity can be reduced up to 10-fold without impairing normal growth [25]–[27]. Previous work has suggested that phylogenetically diverse organisms use this excess reservoir of protein-folding capacity to promote the rapid evolution of new traits through a litany of mechanisms [25],[27],[28]. These include binding and stabilizing mutant proteins with novel activities, promoting the folding and maturation of metastable signal transduction proteins that respond to harsh environmental conditions, and allowing for the release of cryptic genetic variation upon stress. In pathogenic fungi, we have shown that mild compromise of Hsp90 function prevents the *de novo* emergence of resistance to triazoles and, in fact, reverses the resistance of strains of which had previously evolved triazole and echinocandin resistance in the clinic [25],[29],[30]. Hsp90 promotes antifungal drug resistance by stabilizing calcineurin and protein kinase C, two signal transducers that promote resistance by mitigating the stress to the cell wall and membrane that is induced by these drugs [25],[30],[31].

**Figure 2:**
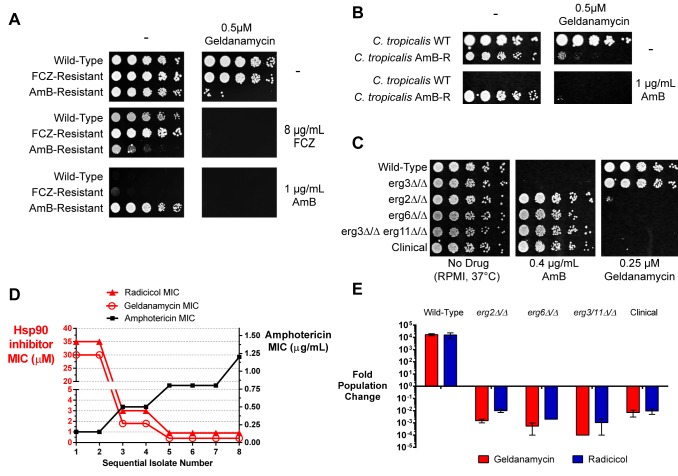
AmB-resistant strains critically depend on high levels of Hsp90 function for survival. (A) Mild inhibition of Hsp90 not only reverses AmB resistance but selectively kills AmB-resistant isolates. Spot assays (5-fold serial dilutions) of wild-type (SC5314), fluconazole-resistant (Isolate #2, [61]), and AmB-resistant (ATCC 200955) *C. albicans* on RPMI media containing antifungals and/or Hsp90 inhibitors at the indicated concentrations. (B) Increased dependence on Hsp90 for AmB resistance is conserved in a *C. tropicalis* clinical isolate. Spot assays of *C. tropicalis* reference strain MYA-3404 (AmB-sensitive) and ATCC 200956 (AmB-resistant) on media containing AmB, geldanamycin, or both compounds. (C) Isogenic, laboratory-generated AmB-resistant mutants also show an increased dependence on Hsp90. Spot assay of laboratory-generated mutants on AmB and geldanamycin. Fluconazole-resistant *erg3* mutant and AmB-resistant clinical isolate are provided for reference. Full MIC data in liquid culture for all strains are provided in Figure S4. (D) *In vitro* selection for AmB resistance leads to hypersensitivity to Hsp90 inhibition. 24-hour MIC80 (drug concentration reducing growth by 80 percent) of geldanamycin, radicicol, and AmB for each isolate from *in vitro* evolution series, tested in YPD at 30°C. Note the discontinuity in the left *y*-axis due to the dramatic decrease in Hsp90 inhibitor MIC. (E) Hsp90 inhibition is cidal to AmB-resistant strains. Viability assays of wild-type and AmB-resistant *C. albicans* strains in the presence of the Hsp90 inhibitors geldanamycin or radicicol. Strains were incubated in liquid culture for 24 h with the drugs and then plated for surviving colony-forming units. Drugs were used at 5 µM in all strains. Error bars indicate SEM; each measurement was performed in duplicate.

We asked if Hsp90 also plays a role in the evolution of resistance to AmB, taking advantage of two structurally unrelated natural products with high specificity for Hsp90 (geldanamycin and radicicol). We examined mutants that had evolved AmB resistance under drug pressure in the clinic (Figure 2A–B), mutants created deliberately by targeting genes in the sterol pathway (Figure 2C), and mutants arising at each step in our in vitro selection (Figure 2D). We spotted drug-resistant and control isolates on media containing no drug, fluconazole, or AmB, in either the presence or in the absence of the Hsp90 inhibitors.

As previously described, modest inhibition of Hsp90 completely blocked fluconazole resistance (Figure 2A, middle panel). It did not affect growth of the fluconazole-resistant isolate in the absence of fluconazole (Figure 2A, top panel). Modest inhibition of Hsp90 also abrogated AmB resistance (Figure 2A, bottom panel). Surprisingly, however, low concentrations of either of the Hsp90 inhibitors completely blocked the growth of all of the AmB-resistant strains, even in the absence of AmB (Figures 2A–D and S4B).

Perturbations in ergosterol biosynthesis can lead to general increases in the accumulation of diverse small molecules. This raised the possibility that the hypersensitivity to Hsp90 inhibitors might simply be due to an increase in their intracellular accumulation. To investigate, we determined the MICs of a panel of seven chemically and mechanistically distinct cytotoxic agents (that do not act through Hsp90) in all of the resistant strains. These MICs were compared to the MICs of geldanamycin and radicicol, as well as two synthetic Hsp90 inhibitors from completely different chemical scaffolds. AmB-resistant mutants were, indeed, generally more sensitive than wild-type cells to many of the cytotoxic compounds. The decrease from the wild-type in the MIC of any of these cytotoxic agents ranged from 2- to 8-fold in the *erg2* or *erg6* mutants and 4- to 16-fold in the *erg3 erg11* mutant (Figure S4A–C). But the decreases in MIC of the four Hsp90 inhibitors were 18- to 48-fold for the *erg2* mutants, 85- to 109-fold for the *erg6* mutants, and 222- to 480-fold for the *erg3 erg11* mutants (Figure S4B–C). Thus, the hypersensitivity of AmB-resistant strains to Hsp90 inhibitors cannot simply be attributed to a general increase in drug accumulation. Rather, the growth of these mutants must critically depend on maintaining very high levels of Hsp90 function even in the absence of AmB.

Was the effect of the Hsp90 inhibitors restricted to growth inhibition, or did they actually cause cell death? We previously showed that Hsp90 inhibition renders the typically cytostatic drug fluconazole cytocidal, killing the fungus instead of simply blocking its growth [29]. Indeed, low concentrations of Hsp90 inhibitors were cytocidal to the AmB-resistant mutants (Figure 2E). But, once again, in contrast to the fluconazole resistant strains, Hsp90 inhibition killed AmB-resistant cells even in the absence of AmB. Thus, the mutations that confer AmB resistance cause a novel and critical dependence on Hsp90 for the simple maintenance of normal viability.

### AmB-Resistant Strains Exhibit Constitutive Activation of Stress Responses

Hsp90 promotes the maturation of a diverse array of metastable signal transduction proteins, including kinases, phosphatases, and ubiquitin ligases (known as Hsp90 clients) [32]. These function in many stress response pathways. Thus, the simplest explanation for the extreme dependence of AmB-resistant strains on Hsp90 is that these normally nonessential client proteins are required to tolerate the perturbations in cellular homeostasis caused by mutations in ergosterol biosynthetic enzymes.

To investigate, we first tested our AmB-resistant strains for constitutive transcriptional activation of a variety of stress response genes. These include targets of the known HSP90 client calcineurin [30],[33], as well as genes involved in the response to iron starvation or oxidative stress, two stresses tightly linked to membrane sterol homeostasis. To provide a point of comparison, we exposed wild-type strains to external stresses known to induce these responses.

**Figure 3:**
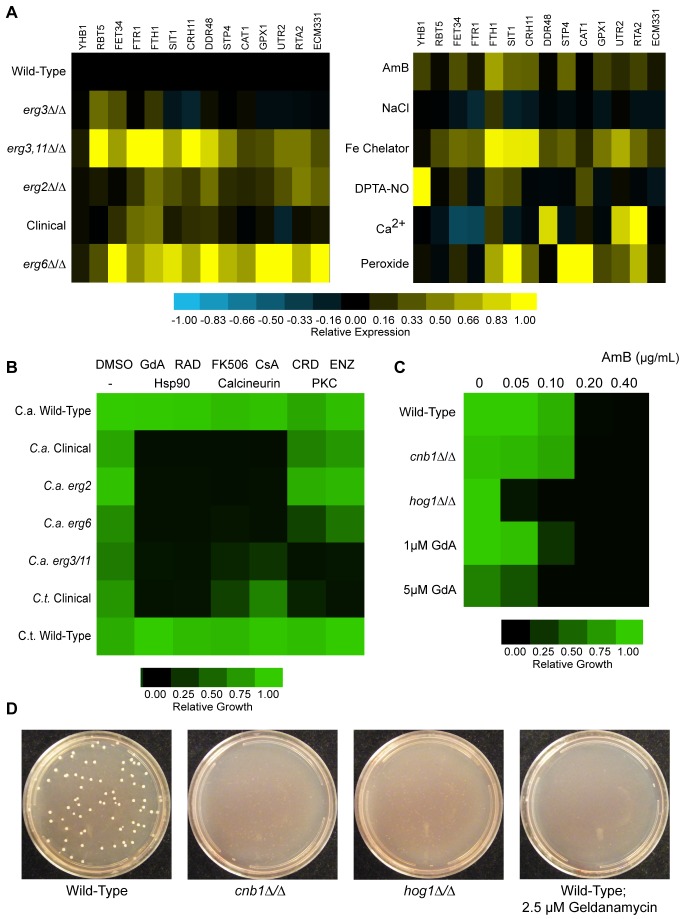
Constitutive stress response activation in AmB-resistant strains. (A) AmB resistant mutants constitutively express diverse stress response genes at high levels in the absence of any external stressors. qRT-PCR profiling of stress response genes in wild-type, fluconazole-resistant (*erg3*), and AmB-resistant mutants of *C. albicans* grown in rich (YPD) media with no added stressors (left panel), compared with wild-type strains treated with a variety of acute stressors known to activate these stress response pathways (right panel). Strains were grown to mid-log phase in YPD, and RNA was isolated by established methods. For stress treatment of wild-type strains (right panel), the indicated stressors are: AmB, 1 µg/mL AmB; NaCl, 0.3 M NaCl; Fe Chelator, 500 µM bapthophenanthroline disulfonate; DPTA-NO, 2 mM DPTA-NONOate; Ca^2+^, 150 mM CaCl_2_; Peroxide, 10 mM tert-butyl peroxide. Expression levels of diverse stress response genes were quantified and normalized to four internal control genes: TDH3, TEF3, ACT1, and RPP2B; the mean value obtained from these four normalizations was used. To generate quantitative comparisons for color visualization, the relative expression level of each gene in each sample was divided by its maximal expression level observed (in any of the deletion strains or stress conditions; see Materials and Methods for further description). (B) Calcineurin and PKC pathways are differentially required for survival of AmB-resistant strains. Growth of AmB-resistant laboratory mutants and clinical isolates, as well as wild-type controls, in the presence of small molecule stress response inhibitors at the following concentrations: Geldanamycin and Radicicol, 2 µM; FK-506, 5 µg/mL; Cyclosporin A, 5 µg/mL; Enzastaurin and Cercosporamide, 5 µg/mL. Growth was assayed after 24 h; values indicate means of duplicate measurements, normalized to wild-type in DMSO. (C) Hog1, but not calcineurin, is required for wild-type levels of AmB tolerance in the absence of ergosterol biosynthesis mutations. Wild-type, *cnb1*, and *hog1* strains, as well as wild-type with 1 or 5 µM geldanamycin, were tested for AmB MIC by microplate dilution. Growth was assayed after 48 h to highlight differences in drug tolerance between strains; values indicate means of duplicate measurements, normalized to the wild type in the absence of AmB. (D) Hog1, Cnb1, and high levels of Hsp90 are required for the *de novo* emergence of AmB-resistant colonies. *ERG2/erg2* heterozygotes from wild-type, *cnb1*, or *hog1* backgrounds were plated at a density of 8×10^6^ cells per plate on media containing 0.4 µg/mL AmB. The wild-type was also plated on media containing AmB and 2.5 µM geldanamycin. Plates were photographed after 2 d.

The AmB-resistant mutants indeed exhibited a constitutive activation of diverse stress responses (Figure 3A, left panel). Pathways of iron starvation were constitutively active, most strongly in the *erg3/erg11* and *erg6* mutants, as evidenced by the high expression of *RBT5* (*ORF19.5636*), *FET34* (*ORF19.4215*), *FTR1* (*ORF19.7219*), and *FTH1* (*ORF19.4802*), and *SIT1* (*ORF19.2179*). Genes responding to general plasma membrane and oxidative stressors also showed generally broad elevation [including *CAT1* (*ORF19.6229*), *GPX1* (*ORF19.86*), *CRH11* (*ORF19.2706*), and *DDR48* (*ORF19.4082*)]. Induction of calcineurin targets [*UTR2* (*ORF19.1671*), *RTA2* (*ORF19.24*), *ECM331* (*ORF19.4255*)] was observed at varying levels as well, most strongly in the various lab strains [33],[34]. The level of constitutive activation of these pathways in AmB-resistant strains was in many cases comparable to levels seen in wild-type strains exposed to severe external stresses (Figure 3A, right panel). Intriguingly, the AmB-sensitive, fluconazole-resistant *erg3* mutant did not show dramatic upregulation of any of the responses tested, but only a weak induction of several iron starvation genes. These data suggest that the mutations that confer resistance to amphotericin concomitantly exert an array of stresses to cellular membrane and redox homeostasis.

### Nonessential Stress Responses Become Essential in AmB-Resistant Strains

Hsp90-dependent stress-response pathways are not essential for growth of wild-type strains. In pathogenic fungi, validated Hsp90 clients include calcineurin, the MAP-Kinase Hog1, and Protein Kinase C (PKC) [30],[31],[35]. To test whether they become essential in the resistant mutants, we took advantage of the high conservation of these proteins: highly selective drugs targeting their human homologs have been developed for diverse therapeutic purposes, and these are active on the fungal proteins as well. These chemical probes allowed us to selectively reduce the activities of these proteins in the genetically intractable clinical isolates. In laboratory strains, these compounds allowed us to bypass the difficulties inherent in maintaining mutations expected to exhibit synthetic lethalities.

To inhibit calcineurin, we used FK-506 and Cyclosporin A, two structurally and mechanistically distinct inhibitors of the phosphatase. To inhibit PKC, we used enzastaurin, a synthetic PKC inhibitor with high selectivity for this kinase, and confirmed our findings with cercosporamide, a natural product fungal-specific inhibitor of PKC [31],[36]. Treatment with either calcineurin inhibitor inhibited growth of all of the Amphotericin-resistant strains, with complete growth inhibition of *erg2* and *erg6* mutants (Figure 3B). The *erg3 erg11* strains were slightly less sensitive to calcineurin inhibitors, but showed a dramatically increased sensitivity to PKC inhibition.

We also asked if wild-type cells rely on Hsp90-dependent stress responses to defend themselves from the toxic effects of AmB. To do so, we created genetic knockouts of these normally nonessential genes in a wild-type background. Indeed, *hog1* mutants were hypersensitive to AmB, while strains lacking calcineurin [*cnb1(orf19.4009)*] were not (Figure 3C). Mild inhibition of Hsp90, which is sufficient to impair calcineurin activity, did not change the AmB MIC. However, more extensive inhibition of Hsp90, which would destabilize Hog1 [35], did sensitize cells to the antifungal. Thus, Hog1 is required to tolerate the stress imposed by drug treatment, while the calcineurin and PKC pathways are required to tolerate the stress imposed by resistance mutations.

Next, we tested the role of Hsp90, Hog1, and calcineurin in the *de novo* emergence of AmB resistance. To do so, we generated *ERG2/erg2Δ* heterozygotes in a wild-type background and in strains lacking Hog1 or calcineurin. We then selected for loss of the remaining allele of *ERG2* by plating on media containing AmB. As expected, *ERG2/erg2Δ* heterozygotes that were otherwise wild-type produced resistant colonies at a rate of ∼10^−5^ (Figure 3D). Low concentrations of the Hsp90 inhibitor geldanamycin completely eliminated the emergence of such colonies. Strains lacking calcineurin also failed to produce resistant colonies, and *hog1* strains produced only a few small, slow-growing colonies. We conclude that Hsp90-dependent stress responses are required to enable the *de novo* emergence of AmB resistance.

### AmB-Resistant Mutants Are Hypersensitive to Stresses Encountered in the Host

Although we have successfully validated the ability of several ergosterol biosynthesis mutations to confer resistance to AmB *in vitro*, resistance rarely evolves during the treatment of infected patients. We wondered if the phenotypic benefit of AmB resistance might be undermined by fitness costs imposed by the mutations. That is, the high levels of internal stress that burden the AmB-resistant mutants might make them unable to withstand the additional external stresses imposed by the host. To investigate, we tested the ability of the resistant mutants and wild-type control to tolerate a range of stresses encountered in host environments, including (1) elevated temperatures (fevers are a universal response to systemic fungal infection and a common side-effect of AmB treatment); (2) hydrogen peroxide, hypochlorous acid, and nitric oxide (used by neutrophils to kill *Candida*); and (3) serum, iron deprivation, and antimicrobial peptides, as these are other common sources of stress in the host.

**Figure 4:**
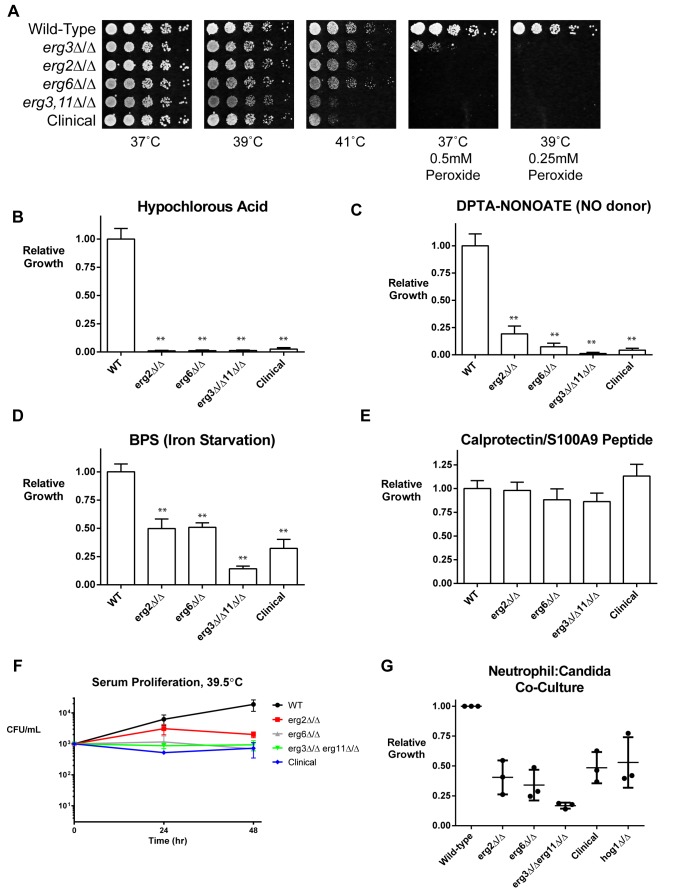
AmB-resistant strains are hypersensitive to the stresses of the host environment. (A) AmB-resistant strains are sensitive to very high febrile temperatures and extremely sensitive to oxidative stress, especially at elevated temperature. Wild-type and AmB-resistant strains were spotted by serial dilution on RPMI media with or without tert-butyl peroxide at the concentrations indicated. (B–E) AmB-resistant strains are hypersensitive to stresses encountered in the host environment. Wild-type and AmB-resistant strains were grown in RPMI media containing 2 mM of hypochlorous acid (B) or 4 mM of the nitric oxide donor DPTA-NONOATE (C), two neutrophil-secreted products that are the critical final effectors of anti-*Candida* immunity. Sensitivity to iron deprivation was tested by growth in RPMI+500 µM of the iron chelator bathophenanthrolinedisulfonic acid (D). No increase in sensitivity to the antimicrobial peptide Calprotectin (10 µg/mL) (E) was observed. Growth values were obtained by normalization to wild-type growth in the same condition. All mutant strains were significantly more sensitive than wild-type to the tested concentrations of hypochlorous acid, DPTA-NONOate, and BPS at 37°C (**p<0.01, two-tailed Student's *t* test), but not to calprotectin. Values indicate the mean of two independent experiments of three replicates each; error bars indicate SEM. (F) AmB-resistant strains proliferate more slowly than wild type in serum at elevated temperature. Wild-type and AmB-resistant strains were inoculated in 100% fetal bovine serum at 39.5°C, and viable colony-forming units were determined by plating dilutions on YPD after 24 and 48 h. Error bars indicate SEM. (G) AmB-resistant strains are hypersensitive to killing by neutrophils. Human neutrophils were isolated from whole blood (see Materials and Methods) and activated by treatment with recombinant TNF-α. *Candida* were added to wells containing neutrophils at a 1∶1 effector∶target ratio and incubated at 37°C for 6 h, at which point neutrophils were lysed and *Candida* growth was quantitated with Alamar blue. Percent growth for each strain was calculated as the fraction of growth in the presence of neutrophils to growth in the absence of neutrophils; mutants were normalized to growth of the WT on each plate. The experiment was performed with a total of three biological replicates from two separate days; error bars indicate mean and SEM.

While AmB-resistant strains grew similarly to wild-type strains at 37°C and 39°C, they grew more poorly at 41°C (Figure 4A). Resistant mutants were hypersensitive to the presence of peroxide, hypochlorous acid, and the nitric oxide donor DPTA-NONOate (Figure 4A–C). Resistant mutants also proliferated moderately more slowly than wild-type cells in the presence of an iron chelator (Figure 4D). Resistant strains were also sensitive to growth in 100% bovine serum (Figure 4F) at elevated temperature, but were not more sensitive to the neutrophil-associated antimicrobial peptide Calprotecin/S100A (Figure 4E).

Next, we tested their susceptibility to attack by neutrophils, the most critical component of the innate immune system in combating acute fungal infection. We isolated human neutrophils from whole blood to 99% purity and activated them by treatment with recombinant TNF-α. Wild-type or AmB-resistant *C. albicans* strains were co-cultured with neutrophils for 6 h, at which point neutrophils were lysed and fungal growth was measured. The *hog1* mutant, previously reported to be hypersensitive to neutrophil attack [37],[38], was included as a positive control. AmB-resistant mutants were indeed significantly hypersensitive to neutrophil attack, exhibiting at least as strong of a defect as the *hog1* mutant (Figure 4G).

### AmB-Resistant Mutants Are Defective in Filamentation and Tissue Invasion

**Figure 5:**
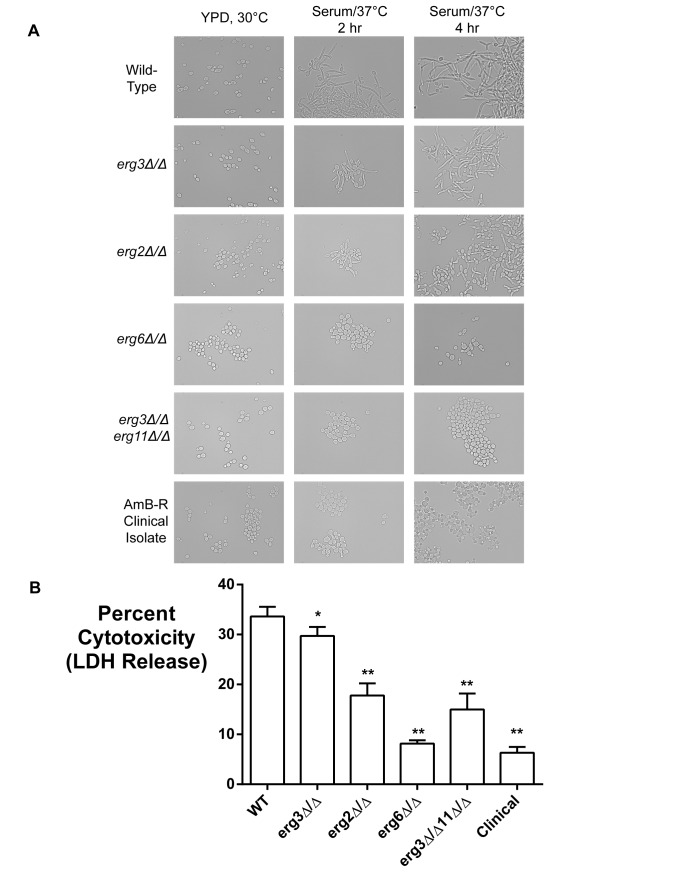
AmB-resistant strains are defective in filamentation and tissue invasion. (A) AmB-resistant mutants fail to properly induce filamentous growth upon stimulation. Wild-type strains and resistant mutants were grown in YPD and then inoculated into RPMI media containing 10% fetal bovine serum at 37°C. Strains were analyzed by DIC microscopy after 2 and 4 h. (B) AmB-resistant strains cause much less damage to endothelial monolayers than wild type. Monolayers of HUVECs (human umbilical vein endothelial cells) were established and infected with *C. albicans*. After 6 h, endothelial cell cytotoxicity was assayed by quantifying LDH release, using uninfected cells as a negative control and cells lysed with 1% Triton X-100 as 100% lysis control. Data were pooled from two independent experiments with six replicate wells each; error bars indicate SEM. All mutant strains were significantly less cytotoxic than wild type (*p<0.05, **p<0.01, two-tailed paired Student's *t* test).

Another potential fitness cost of AmB-resistance mutations could be a compromise of pathogenic virulence mechanisms. *C. albicans* responds to several stimuli in the host environment by undergoing dramatic morphological changes, including the adoption of filamentous hyphal forms. Filamentation enables penetration and invasion of host tissue, and is a highly validated virulence factor for this pathogen [39],[40]. We asked if our AmB-resistant mutants can filament effectively when exposed to 10% fetal bovine serum in RPMI culture media. After 4 h of incubation, wild-type strains exhibited long and robust filaments (Figure 5A). The fluconazole-resistant *erg3* mutant exhibited a mild delay in hyphal protrusion but still formed substantial filaments. However, the *erg2* mutant could only form short and amorphous filaments. The *erg6* and *erg3 erg11* strains and clinical isolates were entirely unable to form hyphal extensions, and remained mainly in the yeast form.

We then asked if this defect in filamentation reduced the capacity of the pathogen for tissue invasion. Monolayers of primary human endothelial cells were established in culture, and infected with the *C. albicans* strains. Lysis of the endothelial cells was monitored by assaying the release of cytosolic lactate dehydrogenase (LDH). All amphotericin-resistant mutants showed dramatic defects in their ability to damage the monolayer (Figure 5B).

### AmB-Resistant Mutants Are Avirulent in Mice

Finally, we compared the virulence of our AmB-resistant laboratory and clinical strains with that of wild-type and fluconazole-resistant (*erg3*) strains in a mouse model of *Candida* fungemia. To provide a rigorous test, we used a relatively high intravenous inoculum of 4×10^6^ fungal cells in young Balb/c mice. At the time of sacrifice, both kidneys were isolated from each mouse. One was analyzed for fungal burden by homogenization and plating of CFU; the other was submitted for histological analysis.

**Figure 6:**
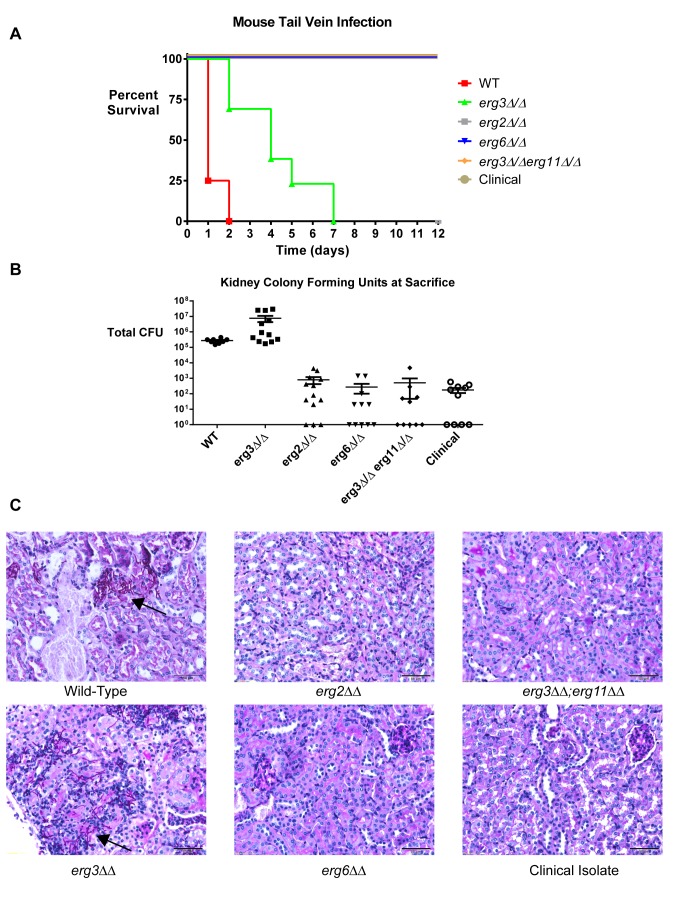
AmB-resistant strains are avirulent in a mammalian model of disseminated candidiasis. (A) AmB-resistant mutants do not cause morbidity even when injected into mice at a high inoculum. Wild-type, *erg3*, and AmB-resistant mutants were grown into log phase for 5 h in YPD, counted, and 4×10^6^ cells of each strain were injected into the tail vein of 7–9-wk-old Balb/c mice (*n* = 8–14 mice per strain). Mice were monitored for weight loss and sacrificed after a >20% drop in body weight or appearance of morbidity. All surviving mice were sacrificed after 12 d. (B) AmB-resistant strains are unable to colonize the mouse kidney. Colonies were counted after homogenization and plating in duplicate of viable *Candida* from one kidney of each infected mouse at the time of sacrifice. As wild-type, *erg3*, and other mutants survived for different periods of time before sacrifice, direct comparisons cannot be made between these groups based on CFU values. However, the extremely low CFU values from AmB-resistant strains are typically indicative of sterilization. (C) AmB-resistant strains do not damage the mouse kidney. Kidneys from each infected mouse were fixed and stained with periodic acid-Schiff stain to visualize *Candida* and kidney pathology. Wild-type and *erg3* strains demonstrated filamentous growth and severe kidney pathology, which was even greater in the *erg3* strain. Examples of sites where fungi are observed are highlighted with a black arrow; fungi appear as long filaments with a purple color. No viable *Candida* were seen in sections from mice infected with AmB-resistant strains. Scale bar, 50 µm.

The wild-type strain killed all infected mice within 1–2 d (Figure 6A). Necropsy revealed high viable fungal burden in the kidney, extensive filamentous fungal morphology, and moderate tissue damage (Figure 6B–C). The *erg3* mutant demonstrated reduced virulence as previously reported [41]–[43], but still killed all mice in an average of 3–4 d. At the time of death, mice infected with this mutant had extremely high kidney fungal burdens, filamentous fungal morphology, and extensive kidney necrosis.

All AmB-resistant mutants, including the clinical isolate, were completely avirulent. Some mice showed mild weight loss in the first day after inoculation. But within a few days all infected mice recovered and appeared healthy. Kidney fungal burdens at 12 d postinoculation were at least three orders of magnitude lower than those of mice infected with wild-type *Candida* or the fluconazole-resistant strain (Figure 6B). Thus, the resistant strains failed to tolerate the host environment or immune attack and colonize this organ, let alone damage it. Histological analysis demonstrated healthy kidneys with some signs of resolving acute inflammation, suggesting that innate immune attack may have contributed to the clearance of these strains (Figure 6C). The lack of morbidity in mice infected with the clinical isolate suggests that this strain was recovered from the patient harboring it not because it was virulent but simply because it had survived AmB treatment.

## Discussion

The emergence of drug resistance has diminished the utility of nearly every class of antimicrobial drug. Yet, 50 years after its introduction, AmB remains as effective as ever. The failure of fungi to evolve resistance to AmB presents a considerable evolutionary puzzle with important consequences for human health. Our work offers a mechanistic solution. In a comprehensive search for mutations that can produce AmB resistance, we sequenced rare resistant clinical isolates, evolved resistant strains in the laboratory, and targeted candidate genes by site-directed recombination. Every mutation that was capable of conferring robust AmB resistance came at great cost to the pathogen. They all diminished *Candida*'s ability to survive the diverse array of stresses that are inherent to growth in a mammalian host, crippled a major virulence factor required for invasive disease (filamentation), and eliminated the capacity to kill mice.

Certainly, AmB therapy often fails, but not because the fungus acquires resistance to the drug [44],[45]. Instead, treatment failure is linked to other factors, including the inability of the drug to penetrate certain niches of the body, dose-limiting renal toxicity, or the underlying disease of the patient [6],[7],[9]. While a small number of AmB-resistant clinical isolates have been reported in large surveys of clinical strain collections, we suspect that many (if not all) of these will prove to be avirulent, as was the one we tested. That is, they may have survived in a superficial niche less exposed to the stresses of the bloodstream but would not be capable of mounting a virulent systemic infection. Such strains might persist in patients with extreme immune system deficiencies. However, resistance to AmB is rare even in patients receiving myeloablative therapies that eliminate immune function [6],[7]. Even in the absence of stress from the immune system, the lack of filamentation and hypersensitivity to other aspects of the host environment (such as fever or iron deprivation) likely restricts the virulence of resistant strains.

Certainly, other explanations have been put forth for the rarity of AmB resistance and may also be relevant. For example, drugs that target lipids are not susceptible to resistance caused by substitutions in drug-binding pockets, a common occurrence with drugs that target proteins. In addition, because AmB acts on the plasma membrane, it is not susceptible to resistance mediated by increased drug efflux. Nevertheless, our work indicates that several deletion or loss of function mutations can readily arise in *Candida* and confer resistance to AmB *in vitro*. But these mutants do not become prevalent in the clinic. It might be argued that AmB resistance is rare because it is dosed intravenously and is not often employed in the types of long-term prophylaxis that breed resistance. However, the structurally similar polyene nystatin, which has the same ergosterol-binding mechanism of action as AmB, is widely used as a topical agent in the prevention of thrush in immunocompromised patients and the treatment of superficial rashes in neonates. Although there has been ample opportunity for resistance to emerge and become a clinical liability, it has not.

Our work also elaborates on the central role played by Hsp90 in potentiating the evolution of new phenotypes, but here it takes on a novel character. As previously reported, Hsp90 plays a critical role in the evolution of drug resistance in *Candida* and *Aspergillus* [25]. Hsp90 allows drug-resistant mutants to survive stresses imposed by triazoles and echinocandins, but is not required to tolerate the mutations conferring resistance to those drugs alone. Such is not the case for AmB. The alterations in sterol structure that confer AmB resistance cannot be achieved without causing a high level of constitutive stress. High levels of Hsp90, then, become essential to simply support viability, even in the absence of the drug. Thus, our findings illustrate yet another way that Hsp90 enables the acquisition of dramatic new phenotypes. Given its conservation, we suggest its role in supporting stress responses operates very broadly in the evolution of phenotypic diversity, allowing organisms to acquire mutations that confer novel phenotypes but simultaneously create stresses that otherwise would not be tolerated.

The clinical experiment of 50 years of AmB use indicates that the emergence of drug resistance, which widely plagues antimicrobial therapeutics, is not inevitable [46]. By elucidating the mechanisms that restrict the evolution of virulent AmB-resistant *Candida*, our work suggests a strategy that might be applied more broadly to prolong the ever-shortening window of efficacy encountered with new antibiotics: the development of compounds that exploit the high costs of resistance mechanisms. This strategy need not require the targeting of lipids. Advances in structural biology and medicinal chemistry have enabled the design of enzyme inhibitors that are less susceptible to resistance mediated by point mutations or by drug efflux [47]–[50]. Resistance to these agents may require the microbe to make more complex changes to its physiology and these, too, may come at a high cost. How might we discover targets that could induce such constraints upon resistance? One possibility is to focus on essential genes that also play critical roles in stress responses or virulence processes, for which rewiring of pathways may fundamentally alter pathogenicity.

In any case, fungal-selective inhibition of Hsp90 presents an attractive mechanism to prevent the emergence of drug resistance to all three antifungal classes in clinical use. Rooted in ancient and conserved biological processes, a similar strategy may prove useful in cancer, where resistance has greatly limited the efficacy of targeted therapeutics. Indeed, pharmacological inhibition of Hsp90 is now being explored as a strategy to forestall the emergence of resistance in diverse malignancies [28]. Investigating the mechanisms that support rapid evolutionary change with an eye to the constant challenges that cells face in their host environment presents a problem of broad biological interest with important clinical implications.

## Materials and Methods

### Ethics Statement

All animal protocols were conducted in accordance with the Guide for the Care and Use of Laboratory Animals of the National Institutes of Health. All animals were maintained according to the guidelines of the MIT Committee on Animal Care (CAC). These studies were approved by the MIT CAC (protocol #0312-024-15). All efforts were made to minimize suffering.

### Media and Growth Conditions

*C. albicans* and *C. tropicalis* strains were routinely maintained at 30°C in YPD (2% Bacto peptone, 2% dextrose, 1% yeast extract). Stocks were maintained in 15% glycerol at −80°C. For generation of deletion mutants, transformants were selected on synthetic medium (2% dextrose, 0.67% Difco yeast nitrogen base with ammonium sulfate) with an amino acid dropout mixture. RPMI 1640 media (Gibco) was buffered with 165 mM MOPS, pH 7.0, and supplemented with 2% dextrose.

### Whole Genome Sequencing, Alignment, Mapping, and Variant Calling

Using an Illumina HiSeq platform with paired-end reads, we obtained an average coverage of 50-fold. After quality control filtering, reads from each sequenced genome were aligned against the *Candida albicans* SC5314 reference sequence, unless otherwise specified (Assembly 21, downloaded from the Candida genome project on June 27, 2011, available here: http://www.candidagenome.org/download/sequence/C_albicans_SC5314/Assembly21/archive/C_albicans_SC5314_version_A21-s01-m01-r03_chromosomes.fasta.gz) using the BWA aligner [51]. This was followed by variant calling with respect to this *Candida albicans* reference using the UnifiedGenotyper from version 1.0.5974 (the version we used throughout these analyses) of the Genome Analysis Toolkit (GATK) [52]. (To ensure that lower quality SNPs that are present in both a parental and derived strain were correctly identified as being common, we disabled the maximum deletion fraction in the call to the UnifiedGenotyper module. The specific parameters used were: “-dcov 1000 -stand_emit_conf 10.0 -stand_call_conf 50.0 –max_deletion_fraction 1.0.”) For the *Candida tropicalis* genome (strain OY5), only a preliminary assembly consisting of 24 scaffolds for the reference example, Candida tropicalis MYA 3404, is currently available. We downloaded assembly “ASM633v1” from NCBI (https://www.ncbi.nlm.nih.gov/nuccore?term=GG692395:GG692418[PACC] on December 20, 2012). Alignment and SNP calling for the *C. tropicalis* genome was performed as per the *C. albicans* genomes. All reads will be available at NCBI under BioProject accession numbers PRJNA194436 (http://www.ncbi.nlm.nih.gov/bioproject/194436) for *C. albicans* and PRJNA194439 (http://www.ncbi.nlm.nih.gov/bioproject/194439) for *C. tropicalis*; the umbrella project accession number is PRJNA195600 (http://www.ncbi.nlm.nih.gov/bioproject/195600).

### Finding Unique Strain-Specific Variants

To identify variants (including SNPs and indels) unique to a strain, we compared the “parental” strain to individual “derived” strains. We used a combination of custom code and the GATK's CombineVariants and SelectVariants features to locate, and then rank by quality, the SNPs and indels detected in open reading frames that were present only in derived strains. From the previously generated VCF files for each of the parental and derived strains as described above, the CombineVariants module was used to create a single list of SNPs (specifically we set the module options “-priority” to the name of the derived strain and the option “-genotypeMergeOptions” to “UNIQUIFY”). With this output VCF file, we employed the SelectVariants module to detect variants unique to the derived strain via the option: “-select "set =  = <derived-strain>" ”. Additionally, to find cases where heterozygotes become homozygotes, or vice versa, we again used the SelectVariants by using the intersection feature: “-select "set =  = Intersection" ”. The merged genotype calls within each common SNP were then further filtered to find high-quality calls where the zygosity changed. Alignments of the reads for the ranked SNPs and indels were then visually inspected in the Integrative Genomics Viewer (IGV) for quality control [53]. In the case of the clinical isolates, we did one pairwise comparison: between the SC5314 wild type and the ATCC 200955 AmB-R clinical isolate (“derived”). For the in vitro selection experiments, we defined the first isolate (#1) as parental, and compared the subsequent three serially derived isolates (#2, #3, #4) back to this parental isolate.

### Loss-of-Heterozygosity (LOH) Visualization

To visualize the LOH events in the in vitro selection series (BV01-BV05), we performed multisample SNP calling using the GATK UnifiedGenotyper module to generate a VCF file containing all SNPs in all four strains (UnifiedGenotyper options: “-glm SNP -nt 1 –downsample_to_coverage 100000”). Following quality filtering of SNPs, from this VCF file, we created a Python script to generate a list of positions of SNPs (relative to reference), if present in any of the four strains. For each SNP position in each of the strains, we were then able to classify whether it was homozygous for the reference base (blue), homozygous for the variant base (red), or heterozygous (white). The resulting “heterogram” for all chromosomes was visualized using the quilt.plot function from the R “fields” package [54]. Regions where a LOH event is likely to have occurred show up as blocks of blue and red SNPs (regions of high homozygosity) against the backdrop of white (heterozygous SNPs).

### Sliding Window Heterozygosity Analyses

For each SNP in a given strain, we extracted the counts of reads containing the reference and variant base from the allele depth (“AD”) VCF annotation at that position. Using only SNPs of quality 1,000 or more, we then computed the “base ratio” at each position by dividing the count of reads for the minor allele base by the total number of reads for both bases at that SNP position, resulting in values of between 0 (complete homozygosity) and 0.5 (complete heterozygosity). We then averaged these base ratios over a 1 kb sliding window for each chromosome. We performed these analyses for the in vitro evolution series (BV01-BV04) of *C. albicans* as well as the single *C. tropicalis* isolate (OY5).

### Spectrophotometric Analysis of Membrane Ergosterol Content

Sterols were extracted and analyzed as previously described [55]. Equal weights of cell pellets were used across strains. Absorption patterns were recorded by scanning between 240 and 300 nm at 0.5 nm intervals.

### Strain Construction

Strains and primers are listed in Tables S1 and S2. Deletion strains were constructed as described in [56], using HIS1, LEU2, and ARG4 markers (the URA3 marker was not used, and all strains used were URA3 wild type). Briefly, PCR products containing approximately 350 nucleotides of upstream and downstream homology for each gene were generated, and fusion PCR was used with a selectable auxotrophic marker to generate the knockout construct. Proper insertion of the auxotrophic marker and loss of the endogenous gene were confirmed by PCR. Two heterozygotes were selected from each initial knockout transformation for the knockout of the second allele, from which two knockout strains were tested for each (four total strains). The *erg3 erg11* mutant was constructed as previously described [21]. These four strains were compared for phenotypic concordance in filamentation and stress resistance to minimize the effect of secondary mutations; additional data on a second mutant strain for key mutants is presented in Figure S5.

### Minimum Inhibitory Concentration and Growth Assays

Antifungal susceptibility was determined in flat bottom, 96-well microtiter plates (Costar) using a broth microdilution protocol as described [25]. Overnight cultures were grown at 30°C in YPD for at least 16 h, and cell density was measured by OD600 before seeding approximately 10^3^ cells per well in YPD or RPMI media, at 30°C or 37°C, as indicated in figure legends. Growth was measured at 24 or 48 h postincubation by alamar blue (Invitrogen) fluorescence with excitation at 550 nm and emission at 590 nm, and in certain cases confirmed by measurement of OD600 after agitation using a spectrophotometer (Tecan). MIC80 was defined as the concentration of drug reducing growth by 80% relative to the wells containing no drug. For susceptibility to AmB and stress response inhibitors (Figures 1D, 3B–C, and S5A), growth scores were determined by normalization of the values for each sample to the values obtained for the wild-type strain in the absence of AmB (or in the DMSO negative control for Figure 3B). For these assays (Figures 1D, 3B–C, and S5A), each condition was tested in duplicate and repeated on at least two different days. Relative growth data were quantitatively displayed in color using Java TreeView 1.1.3 (http://jtreeview.sourceforge.net). Sensitivity to stressors (Figure 4B–E) was similarly determined by microplate dilution and reading of alamar blue dye fluorescence; here, growth scores were calculated by dividing the growth value of the mutant by the growth value of the wild type in each particular stress condition. For stress assays (Figures 4B–E and S5B–C), data represent the mean of six wells, pooled from experiments performed on two separate days. Statistical significance was determined in Graphpad Prism 5.0 using the Student's *t* test function. Error bars represent the SEM for each group. Serum sensitivity was performed as previously described [57].

Cidal and static effects of Hsp90 inhibition were tested generally as previously described [29]. Cells were grown overnight in YPD and diluted to a concentration of 10^4^ cells/mL at 30°C in YPD containing Hsp90 inhibitors at the indicated concentrations. After 24 h, cells were plated onto YPD at two dilutions and colonies counted.

Drugs used in growth assays included AmB (Fungizone, Invitrogen), fluconazole (TCI chemicals), radicicol (A.G. Scientific), geldanamycin (A.G. Scientific), Cyclosporin A (CalBiochem), FK-506 (A.G. Scientific), Cercosporamide (Sigma-Aldrich), and Enzastaurin (LC Labs). All drugs were dissolved in DMSO, with the exception of fluconazole (H_2_0) and AmB, which was obtained as an aqueous suspension with sodium deoxycholate.

### Agar Plate Growth Assays

Spotting assays were performed by growing overnight cultures of strains in YPD at 30°C, washing in PBS, and resuspending in PBS at a concentration of 5×10^6^ cells/mL. Four 5-fold serial dilutions were performed before spotting. All RPMI-agar plates were used within 6 h of pouring due to the potential instability of AmB and peroxides; it is recommended to test a range of AmB concentrations in agar plates due to potential chemical instability. Selection of AmB-resistant colonies was performed using *ERG2*/*erg2Δ* heterozygotes in wild-type, *cnb1Δ/Δ*, or *hog1Δ/Δ* backgrounds on RPMI-agar plates containing 0.4 µg/mL AmB; the wild-type was also selected on media containing AmB and 2.5 µM geldanamycin. Strains were grown overnight in YPD at 30°C, washed in PBS, and plated at a density of 8×10^6^ cells per plate, and incubated for 2 d at 37°C before photographs were taken.

### *In Vitro* Gradual Selection of AmB Resistance

ATCC 10231 was grown overnight at 30°C in YPD and 2×10^8^ cells were inoculated into one liter of YPD containing 0.25 µg/mL AmB at 30°C. Cultures were grown shaking for 24–48 h until turbidity was observed; cells were then removed, washed in PBS, and split for freezing glycerol stocks or reinoculation into media with a 2-fold higher concentration of AmB at the same cell density. The process was repeated until the concentration of AmB reached 32 µg/mL. The process was repeated in three independent selections. Strains were then thawed from glycerol stocks and struck to single colonies for future MIC assays.

### Neutrophil Killing Assay

Neutrophils were prepared fresh from the blood of a healthy human donor following standard protocols, using Histopaque 1077 density gradient centrifugation and hypotonic erythrocyte lysis [58]. After isolation, neutrophils were activated by treating with recombinant TNF-α (10 ng/mL). Killing assays were performed essentially as described in [38],[59]. Briefly, neutrophils were co-cultured with log-phase *C. albicans* strains at a 1∶1 ratio, with both cell types at a concentration of 10^4^/mL. Control wells were inoculated in the identical conditions but without neutrophils added. Plates were incubated at 37°C in a humidified incubator for 6 h, at which point neutrophils were lysed by adding one volume of water containing 0.1% Tween-20 and a 1∶40 dilution of Alamar blue; wells were vigorously pipetted up and down. Alamar blue fluorescence was measured after 90 min of incubation at 37°C. Relative growth was measured by dividing values obtained in the presence of neutrophils by those obtained in their absence for each strain. Control wells lacking *C. albicans* were included to verify that this treatment does not quantify growth of the neutrophil cells. Results from three separate plates are shown; growth of each mutant strain is presented as a fraction of the wild-type growth from the same plate. Statistical analysis was performed using paired Student's *t* test in Microsoft Excel.

### Filamentation Assay

Hyphal induction was performed by growing *C. albicans* overnight at 30°C in YPD, washing in PBS, and diluting 1∶100 into RPMI+10% fetal bovine serum at 37°C (Sigma-Aldrich). After 2 or 4 h, cultures were briefly concentrated by centrifugation and visualized by DIC microscopy.

### Endothelial Cytotoxicity Assay

Endothelial cell invasion assays were performed with human umbilical vein endothelial cells (HUVEC's, Lonza) as previously described [60]. Monolayers were infected with *C. albicans* strains at a 1∶1 HUVEC∶fungus ratio and assayed for cytotoxicity with the CytoTox-96 Lactate dehydrogenase assay (Promega) after 6 h of co-incubation. Cytotoxicity was quantitated as the fraction of LDH release relative to a 100% value of wells treated with 1% Triton X-100 and a baseline value of HUVEC cells not infected with *Candida*. Error bars are indicative of the standard error of the mean for each group. Error bars indicate SEM. Results pooled from two experiments are displayed (six replicate wells per experiment). Statistical analysis was performed using unpaired Student's *t* test in Microsoft Excel.

### Quantitative RT-PCR Expression Analysis

For measurement of the expression of stress response genes, strains were grown overnight in YPD 30°C and diluted to OD600 of 0.15 in YPD, then grown for 5 h to mid-log phase and either centrifuged without treatment or subjected to different stresses. For stressed wild-type cells (SN250 strain), the conditions were as follows: AmB treatment with 1 µg/mL AmB for 15 min, Osmotic shock with 0.3M NaCl for 10 min, Nitrosative stress with 2 mM DPTA-NO (Cayman Chemical) for 15 min, calcium shock with 150 mM calcium chloride for 10 min, oxidative stress with 10 mM tert-butyl peroxide for 10 min, and iron chelation with 500 µM bathophenanthroline sulfonate for 4 h. Cultures were centrifuged at 1,500 g for 5 min and quickly flash frozen in liquid nitrogen. Total RNA was isolated with an RNeasy column kit (Qiagen), normalized to equal amounts of total RNA across samples, and reverse transcribed for 120 min with the high capacity reverse transcription kit (Applied Biosystems). qPCR was performed with SYBR green mastermix (Applied Biosystems) on an Applied biosystems ABI7900 thermal cycler, using oligonucleotides described in Table S2. Each measurement was obtained from an average of four wells, including two biological replicates and two technical replicates. Expression analysis was performed by the comparative ΔCt quantitation method, comparing mutant or stress-treated strains to wild-type untreated strains, using normalization to four internal control genes: TDH3, ACT1, TEF3, and RPP2B; the mean value obtained from the four normalizations was used. For representation by heatmap (Treeview), relative expression levels were determined by dividing each value by the maximum expression level for that gene in any tested condition, with the untreated wild-type samples set as the baseline (as certain genes were induced over 50-fold and others were not induced greater than 4-fold in any condition).

### Murine Model of Systemic Infection

We utilized 7–9-wk-old female Balb/c mice from Charles River laboratory (*n* = 8 mice for WT, 10–14 mice for mutant strains). Each strain was tested in two independent experiments (performed at different times), and data were pooled. Strains to be injected were grown overnight in YPD, diluted, and grown for 5 h into mid-log phase at 30°C, then washed twice in phosphate buffered saline (PBS), counted by hemocytometer and plating of dilutions, and resuspended in PBS at a concentration of 4×10^7^ cfu/mL. We used 100 µL of each suspension to inject mice by lateral tail-vein injection. Mice were weighed daily and monitored for signs of morbidity and sacrificed when body weight decreased by more than 20%. Kidneys were removed and either homogenized in PBS and plated for viable colony units (in duplicate) or submitted for fixation and staining with Periodic-acid Schiff stain. A veterinary pathologist was consulted for histological analysis. All experimental procedures were carried out according to NIH guidelines and MIT protocols for the ethical treatment of animals.

## Supporting Information

Figure S1 **Transposon insertion and heterozygosity analysis in AmB-resistant** ***C. albicans*** **.** (A) Insertion of TCA2 retrotransposon into ERG2 locus. Mapping of reads from wild-type (SC5314) and AmB-resistant (ATCC 200955) *C. albicans*. Left, ERG2 locus on chromosome 1; right; TCA2 locus on chromosome 7. Mate-pairs of reads in the AmB-resistant strain in which one mate maps to ERG2 and the other to TCA2 (identified by long insert sizes) are depicted in the lower panel. All screenshots were generated by the Integrative Genomics Viewer (IGV) [53]. (B) Whole-genome heterozygosity analysis of *C. albicans* ATCC 200955. Single-nucleotide-polymorphisms (SNPs) were analyzed for base ratio at each variant site to determine an allelic ratio; the mean heterozygosity value for total heterozygosity is 0.5, and for total homozygosity the value is 0.0. Base ratios were averaged over a 1 kb sliding window for each chromosome.
(TIF)

Figure S2 **AmB-resistance mutations and heterozygosity analysis in AmB-resistant** ***C. tropicalis*** **.** (A) Mutation in *ERG3* changing conserved serine 258 residue to phenylalanine in *C. tropicalis* ATCC 200956. (B) S258 is universally conserved in Erg3 homologs. Alignment of protein sequence surrounding S258 from Erg3 homologs of *C. albicans*, *A. fumigatus* (XP_747563), and *H. sapiens* (BAA33729). (C) 170-nucleotide deletion from *ERG11* of *C. Tropicalis* ATCC 200956, detected as the complete absence of reads mapping to this region of the gene. Unnecessary lines generated by IGV (not representative of data) were removed. (D) Heterozygosity analysis of ATCC 200956; the incomplete assembly of the *C. tropicalis* genome requires the use of smaller sequence contigs.
(TIF)

Figure S3 **Heterozygosity analysis of** ***in vitro*** **–evolved series.** (A) Whole-genome analysis of loss of heterozygosity events across four sequenced isolates from *in vitro*–evolved series (from Figure 1B). A list of all SNP positions in the four strains was compiled and then analyzed for heterozygosity by base ratio (see Materials and Methods). Sites homozygous for the reference base are depicted in blue, homozygous for the variant base in red, and heterozygous in white. The loss of heterozygosity is focused on the left arm of chromosome 3, which contains ERG6. (B) Heterozygosity analysis of chromosome 3 demonstrates loss of heterozygosity in isolate #4. Sliding-window analysis of base ratio along chromosome 3 depicts loss of heterozygosity in the left arm of chromosome 3.
(TIF)

Figure S4 **Minimal inhibitory concentrations of Hsp90 inhibitors and cytotoxic compounds.** (A) Sensitivity of AmB-resistant mutants to diverse cytotoxic compounds. MIC80 of each strain against each compound was determined by microplate dilution assay in YPD at 30°C. (B) MIC80 of strains to four Hsp90 inhibitors in YPD at 30°C. (C) Fold sensitization, relative to wild-type, of mutant strains to cytotoxic agents and Hsp90 inhibitors (Hsp90 inhibitors highlighted in red for clarity). Table indicates the value obtained when the MIC80 for each compound in the wild-type strain is divided by the MIC80 for that compound in that mutant strain.
(TIF)

Figure S5 **Validation of additional mutant strains.** (A) AmB susceptibility of a second, independently generated laboratory deletion mutant in *ERG2*, *ERG6*, or *ERG3* and *ERG11*, performed as described in Figure 1D. (B) Sensitivity of each additional mutant to hypochlorous acid (B) and DPTA NONOate (C), performed and analyzed as described in Figure 4B–C (**p<0.01, Student's *t* test). (D) Filamentation of each additional mutant in response to stimulation by fetal bovine serum at 37°C in RPMI media, performed as described in Figure 5A.
(TIF)

Table S1 **Strains.** List of strains used in this study.
(DOCX)

Table S2 **Oligonucleotide sequences.** Oligonucleotide primer sequences used in the generation of mutants and RT-PCR experiments.
(XLSX)

## Abbreviations

AmB: Amphotericin B
CFU: colony-forming-unit
ERG: ergosterol
FCZ: fluconazole
Hsp90: heat shock protein 90
HUVEC: human umbilical vein endothelial cell
TNF-α: tumor-necrosis-factor alpha, WT, wild-type
