# Supplementary figures and images for: Fitness Trade-offs Restrict the Evolution of Resistance to Amphotericin B
Source: PLoS Biol. 2013 Oct 29;11(10):e1001692. doi: 10.1371/journal.pbio.1001692 (PMC3812114; doi:10.1371/journal.pbio.1001692)

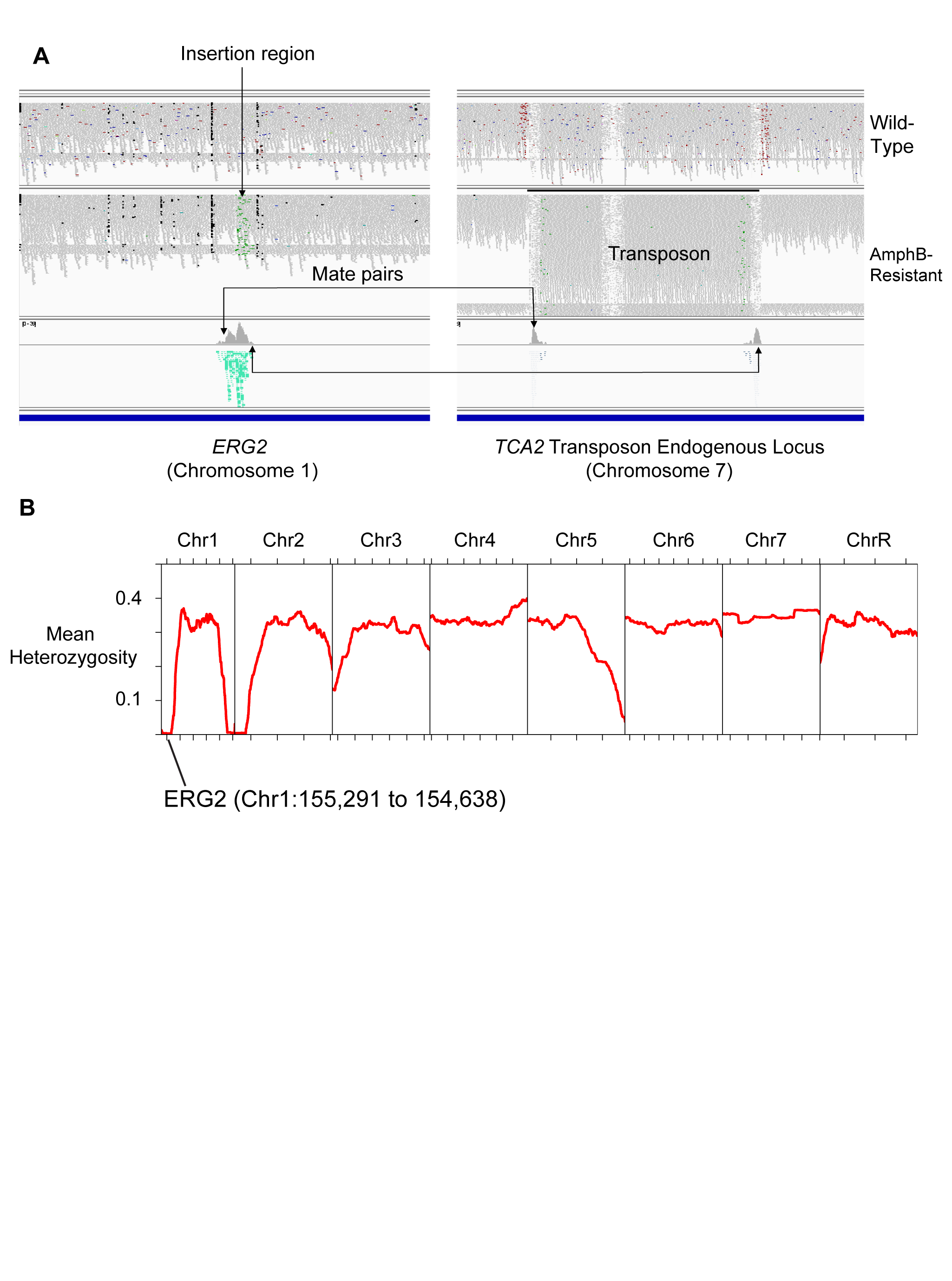

Supplement: Figure S1 — Transposon insertion and heterozygosity analysis in AmB-resistant C. albicans . (A) Insertion of TCA2 retrotransposon into ERG2 locus. Mapping of reads from wild-type (SC5314) and AmB-resistant (ATCC 200955) C. albicans. Left, ERG2 locus on chromosome 1; right; TCA2 locus on chromosome 7. Mate-pairs of reads in the AmB-resistant strain in which one mate maps to ERG2 and the other to TCA2 (identified by long insert sizes) are depicted in the lower panel. All screenshots were generated by the Integrative Genomics Viewer (IGV) [53]. (B) Whole-genome heterozygosity analysis of C. albicans ATCC 200955. Single-nucleotide-polymorphisms (SNPs) were analyzed for base ratio at each variant site to determine an allelic ratio; the mean heterozygosity value for total heterozygosity is 0.5, and for total homozygosity the value is 0.0. Base ratios were averaged over a 1 kb sliding window for each chromosome. (TIF) [file pbio.1001692.s001.tif]

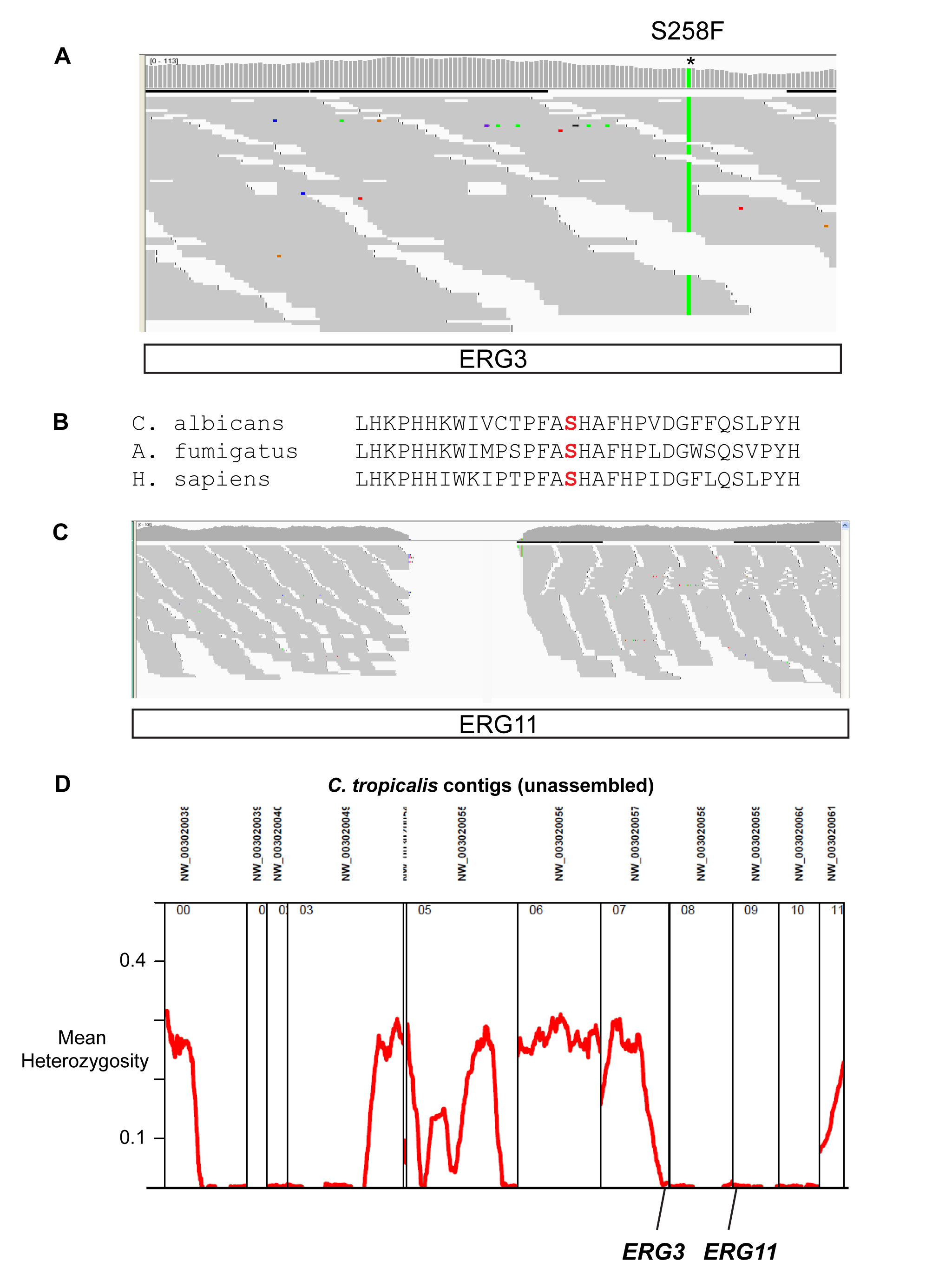

Supplement: Figure S2 — AmB-resistance mutations and heterozygosity analysis in AmB-resistant C. tropicalis . (A) Mutation in ERG3 changing conserved serine 258 residue to phenylalanine in C. tropicalis ATCC 200956. (B) S258 is universally conserved in Erg3 homologs. Alignment of protein sequence surrounding S258 from Erg3 homologs of C. albicans, A. fumigatus (XP_747563), and H. sapiens (BAA33729). (C) 170-nucleotide deletion from ERG11 of C. Tropicalis ATCC 200956, detected as the complete absence of reads mapping to this region of the gene. Unnecessary lines generated by IGV (not representative of data) were removed. (D) Heterozygosity analysis of ATCC 200956; the incomplete assembly of the C. tropicalis genome requires the use of smaller sequence contigs. (TIF) [file pbio.1001692.s002.tif]

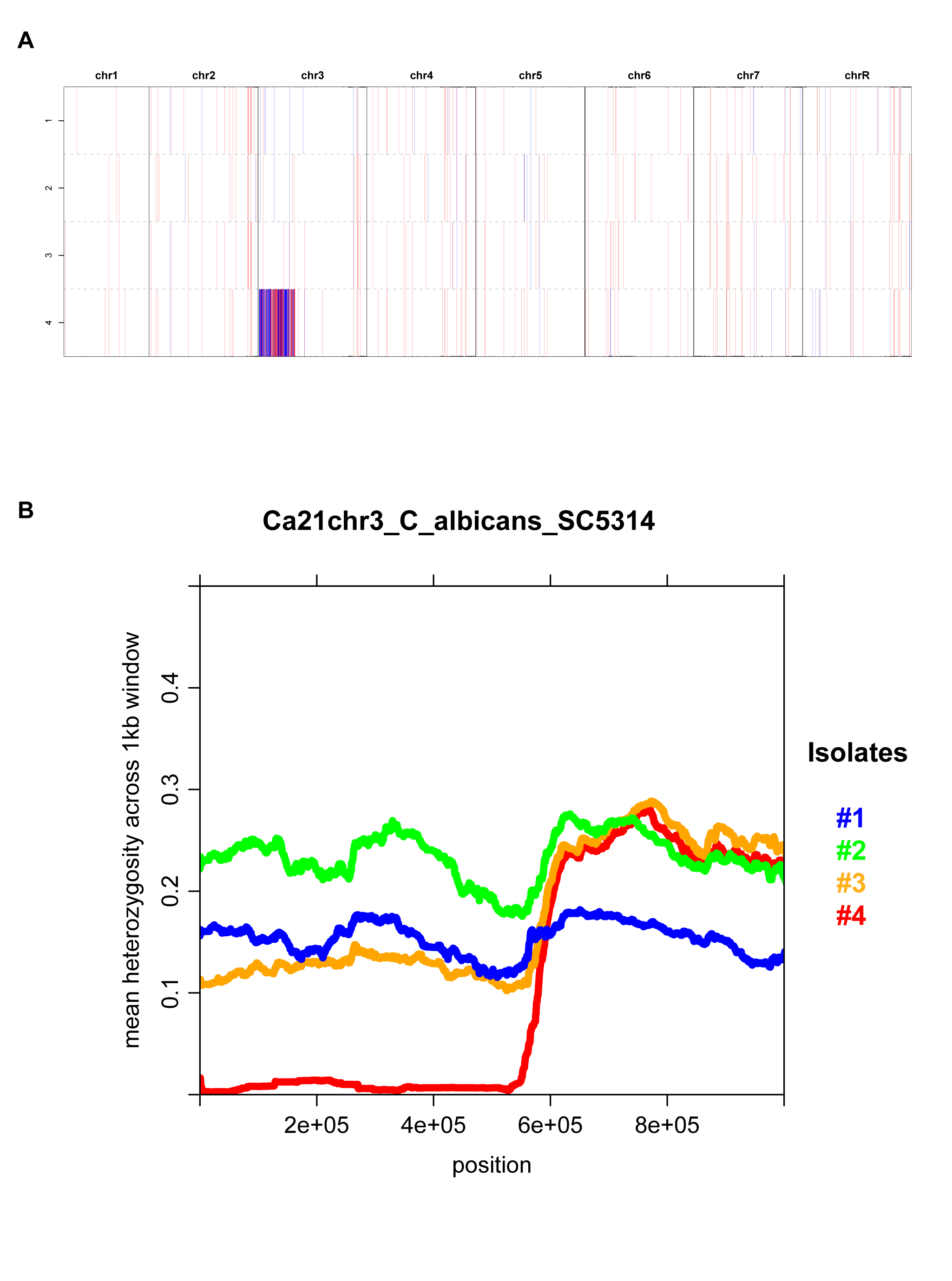

Supplement: Figure S3 — Heterozygosity analysis of in vitro –evolved series. (A) Whole-genome analysis of loss of heterozygosity events across four sequenced isolates from in vitro–evolved series (from Figure 1B). A list of all SNP positions in the four strains was compiled and then analyzed for heterozygosity by base ratio (see Materials and Methods). Sites homozygous for the reference base are depicted in blue, homozygous for the variant base in red, and heterozygous in white. The loss of heterozygosity is focused on the left arm of chromosome 3, which contains ERG6. (B) Heterozygosity analysis of chromosome 3 demonstrates loss of heterozygosity in isolate #4. Sliding-window analysis of base ratio along chromosome 3 depicts loss of heterozygosity in the left arm of chromosome 3. (TIF) [file pbio.1001692.s003.tif]

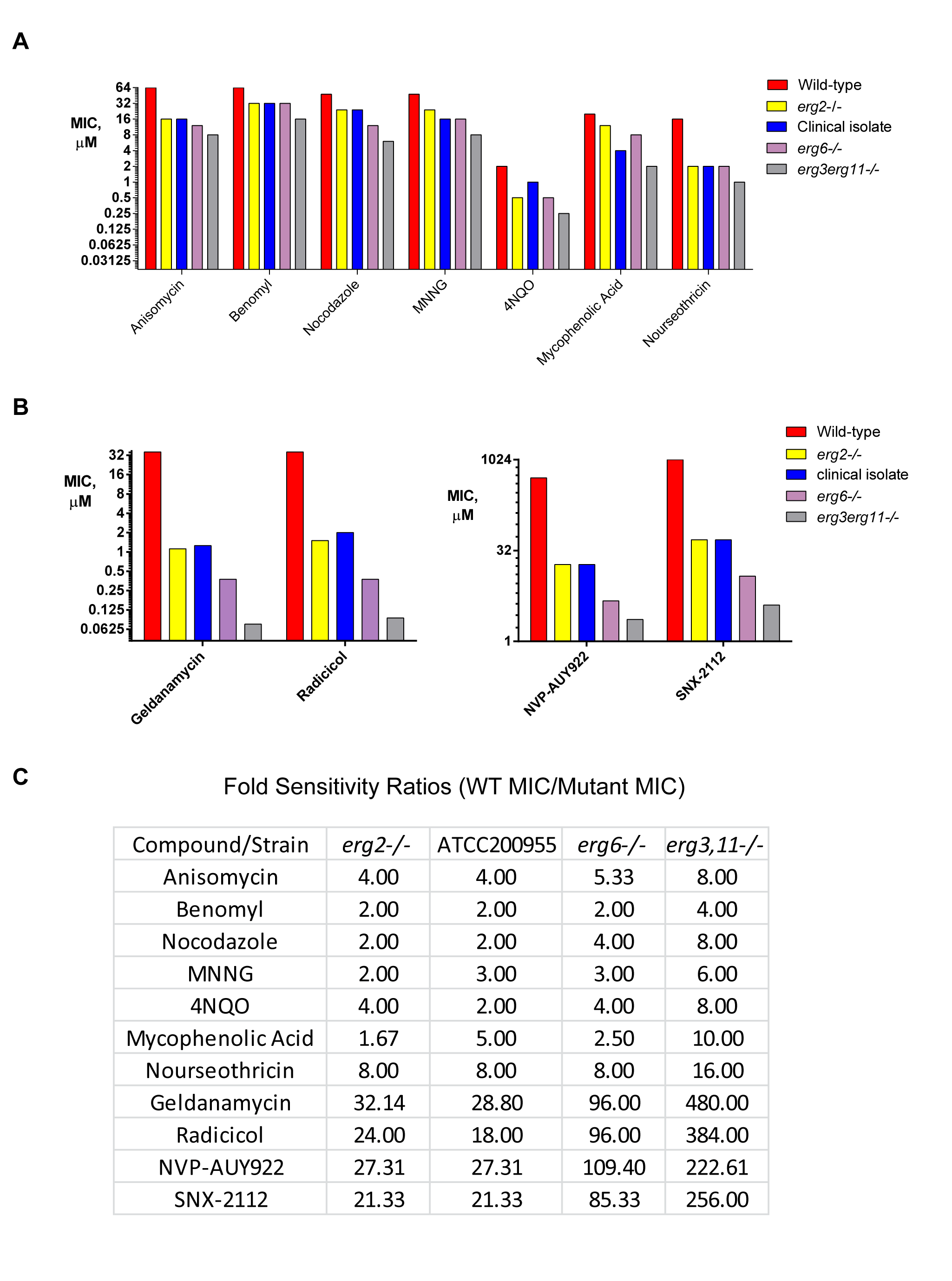

Supplement: Figure S4 — Minimal inhibitory concentrations of Hsp90 inhibitors and cytotoxic compounds. (A) Sensitivity of AmB-resistant mutants to diverse cytotoxic compounds. MIC80 of each strain against each compound was determined by microplate dilution assay in YPD at 30°C. (B) MIC80 of strains to four Hsp90 inhibitors in YPD at 30°C. (C) Fold sensitization, relative to wild-type, of mutant strains to cytotoxic agents and Hsp90 inhibitors (Hsp90 inhibitors highlighted in red for clarity). Table indicates the value obtained when the MIC80 for each compound in the wild-type strain is divided by the MIC80 for that compound in that mutant strain. (TIF) [file pbio.1001692.s004.tif]

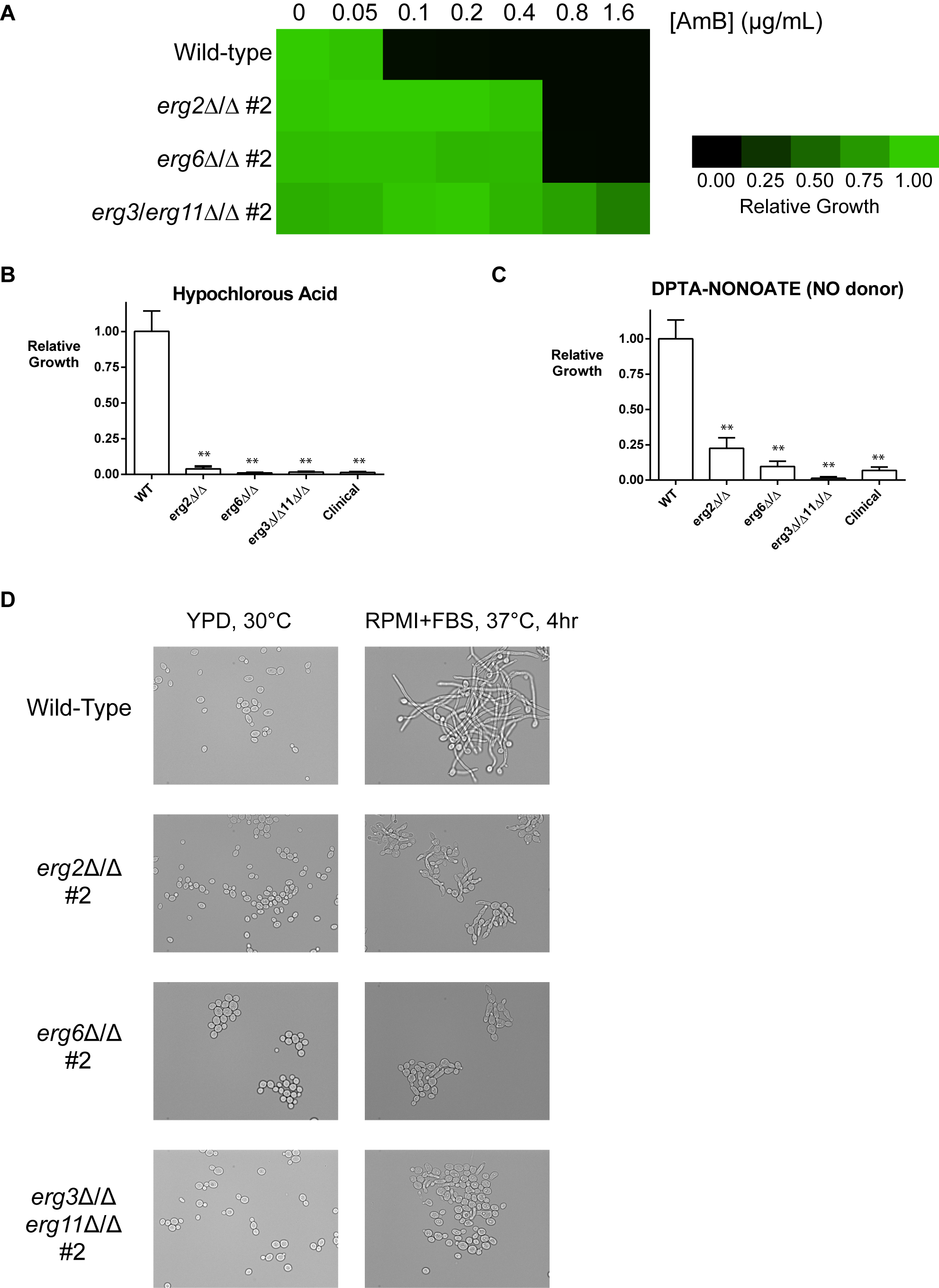

Supplement: Figure S5 — Validation of additional mutant strains. (A) AmB susceptibility of a second, independently generated laboratory deletion mutant in ERG2, ERG6, or ERG3 and ERG11, performed as described in Figure 1D. (B) Sensitivity of each additional mutant to hypochlorous acid (B) and DPTA NONOate (C), performed and analyzed as described in Figure 4B–C (**p<0.01, Student's t test). (D) Filamentation of each additional mutant in response to stimulation by fetal bovine serum at 37°C in RPMI media, performed as described in Figure 5A. (TIF) [file pbio.1001692.s005.tif]
