# Supplementary material for: Fitness Trade-offs Restrict the Evolution of Resistance to Amphotericin B
Source: PLoS Biol. 2013 Oct 29;11(10):e1001692. doi: 10.1371/journal.pbio.1001692 (PMC3812114; doi:10.1371/journal.pbio.1001692)
Supplement: Table S1 — Strains. List of strains used in this study. (DOCX) [file pbio.1001692.s006.docx]

| Strain Name/Name in Text | Genotype | Source |
| --- | --- | --- |
| ATCC 200955 (AmB-R Clinical *C. albicans*) | Prototrophic (naturally *erg2/erg2)* | ATCC |
| ATCC 200956 (AmB-R Clinical *C. tropicalis*) | Prototrophic (naturally *erg3/erg3, erg11/erg11)* | ATCC |
| MYA-3404 (*C. tropicalis* WT reference strain) | Prototophic | ATCC |
| ATCC 10231/*in vitro #1* | Prototrophic | ATCC |
| AmB-R#2/*in vitro* #2 | As ATCC 10231, *erg6* D180G | This study |
| AmB-R#3/*in vitro* #3 | As ATCC 10231, *erg6* D180G (3:1) | This study |
| AmB-R#4/*in vitro* #4 | As ATCC 10231, *erg6* Q70stop/Q70stop | This study |
| Fluconazole-R #2 (CaCi-2) | Prototrophic | White TC, 1997 |
| SN250/Wild-Type | LEU2/leu2Δ HIS1/his1Δ arg4Δ/arg4Δ URA3/ura3Δ::imm^434^ IRO1/iro1Δ::imm^434^ | Noble *et al*, 2005 |
| SN152 | leu2Δ/leu2Δ his1Δ/his1Δ arg4Δ/arg4Δ URA3/ura3Δ::imm^434^ IRO1/iro1Δ::imm^434^ | Noble *et al*, 2005 |
| BV06/*erg2Δ/Δ* | As SN152, *erg2Δ::C.d.HIS1/erg2Δ:C.m.LEU2* | This study |
| BV07/ *erg3Δ/Δ* | As SN152,  *erg3Δ::C.d.HIS1/erg3Δ:C.m.LEU2* | This study |
| BV08/ *erg4Δ/Δ* | As SN152,  *erg4Δ::C.d.HIS1/erg4Δ:C.m.LEU2* | This study |
| BV09/ *erg5Δ/Δ* | As SN152, *erg5Δ::C.d.HIS1/erg5Δ:C.m.LEU2* | This study |
| BV10/ *erg6Δ/Δ* | As SN152, *erg6Δ::C.d.HIS1/erg6Δ:C.m.LEU2* | This study |
| BV11/ *erg3Δ/Δ erg11Δ/Δ* | As SN152,  *erg3Δ::C.d.HIS1/erg3Δ:C.m.LEU2 erg11Δ:C.d.ARG4/erg11Δ/C.d.ARG4* | This study |
| BV12 */erg24Δ/Δ* | As SN152,  *erg24Δ::C.d.HIS1/erg24Δ:C.m.LEU2* | This study |
| BV13/ *cnb1Δ/Δ* | As SN152,  *cnb1Δ::C.d.HIS1/cnb1Δ:C.m.LEU2* | This study |
| BV14/ *hog1Δ/Δ* | As SN152,  *hog1Δ::C.d.HIS1/hog1Δ:C.m.LEU2* | Noble *et al,* 2010 |
| BV16/ WT, *ERG2/erg2Δ* | As SN250,  ERG2*erg2Δ::C.d.ARG4* | This study |
| BV17/ *cnb1Δ/Δ*, *ERG2/erg2Δ* | As SN152,  *cnb1Δ::C.d.HIS1/cnb1Δ:C.m.LEU2;* ERG2/*erg2Δ::C.d.ARG4* | This study |
| BV18/ *hog1Δ/Δ*, *ERG2/erg2Δ* | As SN152,  *hog1Δ::C.d.HIS1/hog1Δ:C.m.LEU2;* ERG2/*erg2Δ::C.d.ARG4* | This study |
|  |  |  |

*C.d., Candida dubliniensis; C.m., Candida maltosa*
